# Editorial on Special Issue “New Era in the Volume Phase Transition of Gels”

**DOI:** 10.3390/gels8070411

**Published:** 2022-06-30

**Authors:** Masayuki Tokita, Masahiko Annaka, Gerald S. Manning

**Affiliations:** 1 Department of Physics, Faculty of Science, Kyushu University, 744 Motooka, Fukuoka 819-0395, Japan; 2 Department of Chemistry, Faculty of Science, Kyushu University, Fukuoka 819-0395, Japan; annaka@chem.kyushu-univ.jp; 3 Department of Chemistry and Chemical Biology, Rutgers University, Piscataway, NJ 08854-8087, USA; jerrymanning@rcn.com

The Special Issue of gels titled “Advancements in Gel Science” has been published from MDPI in 2019. This Special Issue was planned for the 40th anniversary of the discovery of the volume phase transition of gels by the late Professor Toyoichi Tanaka of Massachusetts Institute of Technology. We obtained 16 articles for this Special Issue, and this Special Issue was completed successfully. We, however, learned by editing this Special Issue that there are still many unsolved subjects about the volume phase transition of gels and related areas. We felt that the volume phase transition of gel is still an important subject in the science of gels. We, therefore, planned to launch this Special Issue on the volume phase transition of gels titled “New Era in the Volume Phase Transition of Gels”. Fortunately, we again obtained 11 papers, including both original and review articles. The editorial team thanks all contributors.

It is well known that Tanaka studied the volume phase transition phenomena of gels under the cooperative relationship of theory and experiments and he achieved great success. Although the experimental results on the volume phase transition of gels seem to be explained by the theory established by Tanaka, we know that there were some criticisms even in his time. For instance, the theory based on the analogy with the van der Waals picture of the liquid–gas phase transition is an oversimplification because segments of gel are polymerized into a polymer network in gel. The viscoelasticity due to the polymer network is an essential feature of gels that affects both the equilibrium properties of the gel and the kinetics of the volume phase transition.

The kinetics of the volume phase transition is limited to the linear regime; hence, non-linear effects due to the elasticity of the polymer network is completely neglected. Taking a look at the articles in this Special Issue, we observe that the problems of the volume phase transition of gels raised above were discussed in-depth [1,2,3,4,5,6,7,8,9,10,11]. We hope that this Special Issue will promote a better understanding of the volume phase transition of gels. We thank again all scientists who collaborated with our plan of the Special Issue “New Era in the Volume Phase Transition of Gels”.
